# A Community-Based Study of Enduring Eating Features in Young Women

**DOI:** 10.3390/nu4050413

**Published:** 2012-05-24

**Authors:** Phillipa J. Hay, Petra Buettner, Jonathan Mond, Susan J. Paxton, Frances Quirk, Bryan Rodgers

**Affiliations:** 1 School of Medicine, University of Western Sydney, Locked Bag 1797, Penrith NSW 2751, Australia; 2 School of Medicine, James Cook University, Townsville QLD 4811, Australia; Email: Petra.buttner@jcu.edu.au (P.B.); frances.quirk@jcu.edu.au (F.Q.); 3 School of Public Health, James Cook University, Townsville QLD 4811, Australia; Email: Jonathan.Mond@anu.edu.au; 4 School of Psychological Sciences, La Trobe University, Melbourne VIC 3086, Australia; Email: Susan.Paxton@latrobe.edu.au; 5 Australian Demographic and Social Research Institute, The Australian National University, Canberra ACT 0200, Australia; Email: Bryan.Rodgers@anu.edu.au

**Keywords:** binge-eating, help-seeking, treatment barriers, bulimia nervosa

## Abstract

We conducted a prospective exploration of the temporal course of eating disorder (ED) symptoms in two cohorts of community women. One hundred and twenty-two young women (Cohort 1) identified in a general population based survey with ED symptoms of clinical severity agreed to participate in a 5-year follow-up study. A comparative sample (Cohort 2) of 706 similar aged self-selected college women (221 with disordered eating) was recruited one year later. Both ED groups were given a health literacy package in the first year. ED symptoms, health related quality of life, and psychological distress were assessed annually with the Eating Disorder Examination Questionnaire, the Short Form—12 Health Survey and the Kessler Psychological Distress Scale, respectively. Forty percent (Cohort 1) and 30.3% (Cohort 2) completed questionnaires at each year of follow-up. In both groups, there was early improvement in ED symptoms which plateaued after the first year, and participants retained high EDE-Q scores at 5 years. BMI increased as expected. Mental health related quality of life scores did not change but there were small improvements in psychological distress scores. The findings suggest little likelihood of spontaneous remission of ED problems in community women.

## 1. Introduction

Eating disorders are a common mental health problem [1] and a growing problem worldwide in developed and developing countries such as Australia [2] and India [3]. In addition, disordered eating associated with overweight and obesity is increasing concomitantly [4]. As well as being common, eating disorders are serious mental health problems with important medical co-morbidities. Features include behaviours, such as restrictive dieting, vomiting and compulsive exercise, that aim to address extreme concerns about body shape and weight. Such body image concerns dominate the person’s self-view and are a core preoccupation. People with an eating disorder often have irregular eating patterns, fluctuating between severe food restriction and overeating that becomes out of control (binge eating). Current diagnostic schemes define three Eating Disorders: anorexia nervosa, bulimia nervosa [5,6] and Eating Disorders not Otherwise Specified (EDNOS) [5] or atypical anorexia nervosa or bulimia nervosa [6]. The former are well-defined Eating Disorders, but are much less prevalent than EDNOS in the community or primary care settings [1,7]. In revisions to diagnostic schemes, binge eating disorder (BED), a subset of EDNOS, is likely to become a full disorder [8]. The remainder of EDNOS mostly comprises people whose (i) symptom severity excludes them from a diagnosis of bulimia nervosa or anorexia nervosa, for example they fail to meet the frequency requirement for binge eating episodes, or their binging episodes are not unusually large (subjective binge eating), or (ii) a range of disorders characterised by extreme weight control methods [4,5]. Research has also confirmed that BED and other EDNOS are associated with poor quality of life and impairments in home, work, personal, and social life [9]. Such disorders are the focus of the present paper. 

Whilst there have been many longitudinal studies of outcome in eating disorders these have largely been of clinic or treatment seeking samples [10,11]. However, the vast majority of people with common eating disorders do not seek effective help [12,13] and have BED, other EDNOS or subclinical syndromes [1] and much less is known about their outcomes. In a naturalistic community study, Patton *et al*. [14] followed 107 adolescents with an eating disorder over a 10-year period. The findings suggested partial eating disorders were unlikely to progress to full eating disorders, but were associated with significantly increased psychiatric co-morbidity and persistent underweight. The authors acknowledged that the findings may well differ from those of older samples as disorders such as bulimia nervosa and BED have later age onset in older adolescence and young adulthood. A naturalistic community based 5-year study [15,16] of adult women with BN and BED found that half the BN sample (36 of 74) still met diagnostic criteria for an eating disorder (24 with EDNOS) while only 15% (5 of 34) of those with BED met diagnostic criteria. However, findings from the McKnight longitudinal naturalistic 4-year outcome study of BED [17] indicated only a small proportion of BED recover spontaneously. A recent general population study of adolescents followed into their third decade with disordered eating indicated more persistence of disordered eating particularly when associated with purging [18]. This study did not however examine time trends in psychopathology such as body image or weight and shape concerns.

In this context, the present study was planned to investigate the course of two community cohorts of young women with disordered eating and associated eating disorder psychopathology. The first was recruited from the general population, and the second was a convenience sample recruited from colleges of higher education. Both cohorts were participants in two similar randomised controlled trials (RCTs) aimed to prompt evidence based treatment seeking. In these RCTs at the start of the first year (baseline) the participants were randomised to receive either an eating disorder mental health literacy (ED-MHL) [19] intervention or information about their symptom scores and local mental health services only, with the comparison group (as required by ethical consideration) receiving the intervention at the end of the first year. The intervention comprised a single posted package of information about treatment of BN and related disorders, purchasing information on the book “*Binge Eating and Bulimia Nervosa: A Guide to Recovery*” (Cooper, 1995 [20]) that included a detailed psycho-educational section and a self-directed cognitive-behaviour therapy, recommended websites for further information on treatments, lists and contact details of local eating disorder specialist treatment facilities, and contact details for the (local) eating disorders support group and consumer organisation. At baseline the control participants received information about local mental health services only. 

Early outcomes in these participants were mixed. Although there was improvement in both cohorts it was unclear how much could be ascribed to the ED-MHL intervention [21,22]. At two years eating disorder psychopathology remained high in the first cohort [23]. Despite this, and the known social and personal costs of eating disorders, at four years the majority of participants had not sought or been referred for effective or evidence based therapy [24]. The present paper reports the longer-term, outcomes over the 5-year of both cohorts. Our specific aims were to investigate the persistence or otherwise of eating disorder symptoms, both behaviours and psychopathology, throughout the five years in these participants.

## 2. Method

### 2.1. Participants

#### 2.1.1. Cohort 1

Participants for this study were derived from the Health and Well-Being of Female Australian Capital Territory (ACT) Residents Study, which was an epidemiological study of disability, health-service utilisation and mental health literacy among women with bulimic-type ED’s in the community. A detailed description of the recruitment procedures for that study can be found in Mond *et al.* [25] and the follow-up phases to two years in [23]. Ten thousand women aged between 18 and 42, randomly selected from the ACT electoral roll, were invited to complete a self-report questionnaire. The questionnaire included socio-demographic information, self-reported height and weight, as well as measures of eating disorder symptoms, psychological distress and quality of life. A total of 324 women met screening criteria and were then interviewed to determine ED diagnoses using the Eating Disorder Examination (EDE) [26]. Of these women, 185 were identified with ED symptoms of clinical severity, and 122 consented to participate in a follow-up study, of whom 78 (64%) completed follow-up at the fifth year and 48 (40%) completed assessment at each of the 5 years. Clinical severity was denoted by the presence of current extreme weight/shape concerns and/or current regular (e.g., occurring weekly over the past three months) binge eating and/or any extreme weight control behaviours such as self-induced vomiting and/or laxative/diuretic use and/or fasting or severe food restriction and/or “driven” exercise as determined on the EDE. For example, the EDE defines extreme weight or shape concerns as a level of ≥4 (maximum 6) on the relevant questions.

#### 2.1.2. Cohort 2

This sample was derived from a parallel longitudinal survey of women with disordered eating recruited through advertisements in four universities and colleges of higher education in two Australian States (Queensland and Victoria). Details of the total sample at baseline have been reported in Mond *et al.* [27]. Recruitment strategies varied and included approach via central University email/web mail, printed advertisements in student bulletins and halls of residence and direct approach to students in University common areas. For individuals approached via email, participants were given the option of completing an on-line questionnaire. For other participants, questionnaires were provided in hard copy with reply-paid envelopes. The questionnaire included measures of eating disorder psychopathology and health-related quality of life (as completed by the first sample, see below). The present study cohort of 221 symptomatic women (217, 98% of whom participated in the first year RCT) were selected from the 794 respondents if they had had current extreme weight/shape concerns and/or current regular (e.g., occurring weekly over the past three months) binge eating and/or any extreme weight control behaviours such as self-induced vomiting and/or laxative/diuretic use and/or fasting or severe food restriction and/or “driven” exercise and/or who self-identified on the ED-MHL survey as currently having a problem like that of a fictional women with bulimia nervosa. One hundred and fifteen (52%) completed the fifth year of assessment, and 67 (30.3%) completed assessments are each of the five years. 

### 2.2. Measures

The questionnaire package mailed out to participants each year included demographic information including marital status, employment status, educational level obtained, and self-reported height and weight. At years 1, 2 and 4 the questionnaire also included questions regarding whether participants had sought help during the previous 12 months for any emotional or mental health problem, or for a problem specifically related to eating, and in year 4 a detailed interview regarding help-seeking was conducted [24]. The same measures of eating disorder and psychological variables as used at Time 1 were also included. These were as follows.

Eating Disorder symptoms: The Eating Disorder Examination Questionnaire [28,29] (EDE-Q) was used to examine eating disorder symptoms. The EDE-Q has been validated in community and clinic samples of people with eating disorders. A global score of eating disorder attitudes and restraint, and four sub-scales (*i.e.*, shape, weight and eating concern and dietary restraint) can be derived and it assesses frequency of specific diagnostic behaviours such as binge eating and driven exercise. Driven exercise is defined as having “exercised hard as a means of controlling your shape or weight”. 

Mond *et al.* have reported Australian norms [30]. The four subscales have been found to have good reliability (alpha and test-retest reliability coefficients ≥0.8) and moderate predictive validity in identifying probable cases of the more commonly occurring eating disorders (Se = 0.8, Sp = 0.8, PPV = 0.5) [31]. Cronbach alpha in the present study was 0.77. 

Physical and Mental health: The well-validated 12-item SF-12 [31] measures dimensions of health and role limitations due to physical and mental ill-health, for which component Physical and Mental Health Component summary scales (PCS and MCS respectively) can be derived. Cronbach’s alpha in the present study was 0.82. 

Psychiatric symptoms: General psychiatric symptoms were assessed with the Kessler-10 item distress scale (K-10) [32]. The K-10 has high internal consistency and test-retest reliability. It is designed to detect cases of anxiety and affective disorders in the general population [33]. A score of 16–29 indicates medium risk of the presence of an anxiety or depressive disorder, and 30–50 high risk [34]. Cronbach’s alpha in the present study was 0.91. 

### 2.3. Data Analysis

Numerical data were inspected for normality. Repeated measurement ANOVA was applied to test for differences in eating disorder and other clinical features over the 5 year study period. Mauchly’s test statistic was used to determine whether sphericity could be assumed or was violated. Wilks’ lambda was used in situations where sphericity was assumed; Greenhouse-Geisser test and tests for linearity were used in situations where sphericity was violated. Frequency of binge eating (objective and/or subjective bulimic episodes in past month) and driven exercise were skewed and subjected to logarithmic transformation. Post-hoc differences within repetitions were adjusted for multiple testing by Bonferroni (*t*-tests were accepted as being significant if *p* < 0.0083, α = 0.05/6 = 0.083). Participants who completed all five follow-up assessments (*n* = 48 for Cohort 1 and *n* = 67 for Cohort 2) were compared with people who did not using *t*-tests and the Independent Samples median test. Because of the low prevalence of purging behaviours and little variance (all median and IQ range scores were 0 at all time points in Cohort 2 and all but two time points in Cohort 1) and risk of Type 2 error this behaviour was not reported beyond the baseline and not examined statistically.

### 2.4. Ethics

The research (for both study cohorts) was approved by the Human Research Ethics Committees for James Cook University, La Trobe University and the University of Western Sydney (HREC 07/240), with the last as lead committee. All participants completed informed consent.

## 3. Results and Discussion

### 3.1. The Two Cohorts at Baseline

Both cohorts were predominately EDNOS type with regular binge eating (89 and 79% respectively) but low (less than 10 and 15% respectively) rates of regular purging behaviours. As shown in Table 1, within each cohort when participants at baseline were compared with those who completed all 5-year follow up contacts and those who did not differences were small and not significant for demographic characteristics including age, marital status, dependent children at home, level of education, Australian birth (over 90 and 80% respectively), English as first language, as well as baseline values of outcome measures (the latter shown with age in Table 1, all *p* > 0.05). As expected, each cohort had moderate to high levels of eating disorder symptoms (comparable to those found in clinical samples [25]), psychological distress and impaired mental health related quality of life. 

**Table 1:** Comparative baseline features (mean (SD) or median (IQR)) of those who completed each of the 5 years of follow-up and those who did not.

	Cohort 1		Cohort 2	
	Completers	Non-Completers	Completers	Non-Completers
	*n* = 48	*n* = 74	*n* = 67	*n* = 154
	Mean (SD)			
Age/years	28.6 (5.8)	28.4 (6.6)	26.0 (8.0)	23.8 (7.3)
EDE-Q ^a^ scores				
Global	3.8 (0.9)	3.7 (0.7)	3.3 (1.0)	3.3 (1.2)
Restraint	3.4 (1.4)	3.1 (1.5)	3.1 (1.3)	2.9 (1.5)
Shape concern	4.6 (1.1)	4.8 (0.8)	4.2 (1.2)	4.1 (1.2)
Weight concern	4.1 (0.9)	4.1 (0.9)	3.7 (1.1)	3.8 (1.3)
Eating concern	2.9 (1.4)	2.9 (1.1)	2.3 (1.3)	2.4 (1.5)
Short-form 12 MHC ^b^	37.8 (12.2)	38.2 (10.8)	40.6 (11.2)	38.2 (11.7)
K-10 ^c^	22.1 (8.0)	21.7 (6.0)	22.7 (8.5)	23.3 (8.0)
BMI (kg/m^2^)	27.4 (6.6)	26.7 (7.1)	25.9 (8.5)	23.3 (8.0)
	Median (IQ range)			
Binge eating ^d^	7.5 (2–16)	10.0 (4–20)	8.0 (2–15)	7.0 (1–15)
Driven exercise ^e^	8.0 (0–18)	0 (0–14)	0.5 (0–10)	4.0 (0–15)
Purging ^f^	0 (0–1.5)	0 (0–0)	0 (0–0)	0 (0–0)

^a^ Eating Disorder Examination Questionnaire, Global and subscale scores [28], for comparison community normative values for these in young adult women are respectively Global 1.52 (1.25), Restraint 1.30 (1.40), Eating concern 0.76 (1.06), Weight concern 1.70 (1.51) and Shape concern 2.23 (1.65) [30]; ^b^ mental health component score of the Short form 12 health related quality of life measure [32]; ^c^ Kessler 10 item measure of psychological distress [35]; ^d^ Monthly frequency of objectively large binge eating episodes as defined in the DSM-IV [5] and/or subjective binge eating episodes as defined in the Eating Disorder Examination [26]; ^e^ defined as number of times in the past month the person exercised hard as a means of controlling her shape or weight; ^f^ number of episodes of use of laxatives, vomiting or diuretics as a means of controlling shape or weight.

### 3.2. Changes Each Year in Eating Disorder and Other Features over the 5 Years of Follow-Up

#### 3.2.1. Cohort 1 (See Table 2 below)

Means over the 5-year were compared with one-way repeated measures ANOVA. All were significantly different, *p* < 0.05. Polynomial contrasts indicated significant linear (*p* = 0.011) and quadratic (*p* < 0.005) trends in mean EDE-Q global scores. Polynomial contrasts indicated a significant quadratic trend in terms of EDE-Q shape concern scores (*p* = 0.001). The mean EDE-Q restraint scores showed a significant linear as well as quadratic trend (*p* = 0.001 and *p* = 0.037, respectively). Polynomial contrasts indicated a significant quadratic trend in terms of EDE-Q eating concern scores (*p* = 0.005). Binge eating (subject to logarithmic transformation) and BMI followed a linear trend over the years, (*p* = 0.001). Driven exercise fluctuated over the five years and did not follow a linear or quadratic trend. At four and five years frequency of driven exercise was lower than at baseline but greater than year 1.

Differences in Mental Component Summary Scores (MCS) were not significant (mean 37.8, SD 12.3 at 5 years). Mean K-10 scores were significantly improved with differences significant between baseline and each other year (mean 21.9, SD 7.7 at 5 years, *p* = 0.021).

**Table 2:** Comparative eating disorder features over each of the 5-years for Cohort 1 (*n =* 48).

	Base	Year 1	Year 2	Year 3	Year 4	Year 5
	Mean (SD)					
EDE-Q ^a^						
Global	3.8 (0.8) ^d^	3.1 (1.3)	3.2 (1.2) ^e^	3.1 (1.3) ^e^	3.1 (1.4)	3.2 (1.2)
Restraint	3.4 (1.3) ^d^	2.7 (1.7)	2.9 (1.5)	2.5 (1.6) ^e^	2.5 (1.6)	2.5 (1.7)
Shape concern	4.6 (1.1) ^d^	3.8 (1.5) ^e^	4.0 (1.4)	4.0 (1.6)	3.5 (1.6)	4.2 (1.4)
Weight concern	4.1 (0.9) ^d^	3.4 (1.4)	3.5 (1.2) ^e^	3.6 (1.4)	3.8 (1.7)	3.8 (1.3)
Eating concern	2.9 (1.4) ^d^	2.4 (1.6)	2.2 (1.5)	2.3 (1.5) ^e^	2.5 (1.7)	2.4 (1.5)
BMI (kg/m^2^)	27.4 (6.7)	27.6 (6.3)	28.0 (6.1)	27.3 (5.9)	28.3 (6.7)	28.7 (6.8)
	Median (IQ range)					
Binge eating ^b^	7.5 (2–16)	8.0 (0–16)	4.0 (0–11)	5.0 (0–12)	5.0 (0–13)	4.0 (4–10)
Driven exercise ^c^	8.0 (0–18) ^d^	1.0 (0.3–4) ^f^	10 (0–20) ^d,e^	4.0 (0–15) ^d^	0 (0–12) ^e^	1.5 (0–16) ^e^

^a^ Eating Disorder Examination Questionnaire, Global and subscale scores [28]; ^b^ Monthly frequency of objectively large binge eating episodes as defined in the DSM-IV [5] and/or subjective binge eating episodes as defined in the Eating Disorder Examination [26]; ^c^ monthly frequency; ^d,e,f^ superscripts indicate a significant difference between means.

#### 3.2.2. Cohort 2 (See Table 3 below)

The mean scores for all eating disorder features excepting driven exercise were significantly different over all 5 years (all *p* < 0.01). Polynomial contrasts indicated a significant linear trend for the mean EDE-Q global score (*p* < 0.005). The mean EDE-Q weight concern scores followed linear (*p* < 0.005) as well as quadratic (*p* < 0.005) trends. The mean EDE-Q shape concern followed significant linear and quadratic trends (*p* < 0.005). The mean binge eating (subjective and objective bulimic episodes during the past month subject to logarithmic transformation) scores followed both linear and quadratic significant trends. Polynomial contrasts indicated a significant linear (*p* = 0.001) and quadratic (*p* = 0.025) trend in mean BMI. There were no significant differences over the 5-year for frequency of driven exercise and no linear or quadratic trends.

Differences in the mean SF12 MCS scores were not significant over the years (mean at five years 45.4, SD 10.8). Polynomial contrasts indicated a linear trend. The mean K10 scores were significantly improved over the years with differences between baseline and each other year, between year 1 and years 3 and 5, and between year 2 and 3 (mean at five years 17.9, SD 6.7, *p* < 0.005). 

**Table 3:** Comparative eating disorder features over each of the 5-years for Cohort 2 (*n* = 67).

	Base	Year 1	Year 2	Year 3	Year 4	Year 5
	Mean (SD)					
EDE-Q ^a^						
Global	3.3 (1.0) ^d^	2.4 (1.2) ^e^	2.5 (1.4) ^e^	2.1 (1.3) ^e^	2.2 (1.2) ^e^	2.2 (1.3) ^e^
Restraint	3.1 (1.3) ^d^	2.1 (1.5) ^e^	2.5 (1.4)	2.0 (1.5) ^e^	2.0 (1.5) ^e^	1.8 (1.5) ^e^
Shape concern	4.1 (1.2)	3.1 (1.5)	3.2 (1.5)	2.9 (1.7)	2.8 (1.6)	3.0 (1.7)
Weight concern	3.7 (1.1) ^d^	2.7 (1.4) ^e^	2.8 (1.5) ^e^	2.3 (1.4) ^e^	2.4 (1.4) ^e^	2.6 (1.6) ^e^
Eating concern	2.3 (1.3) ^d^	1.5 (1.3) ^e^	1.6 (1.4) ^e^	1.4 (1.7) ^e^	1.4 (1.2) ^e^	1.4 (1.3) ^e^
BMI (kg/m^2^)	25.9 (6.9)	25.6 (6.0)	25.9 (6.5)	26.6 (7.1)	27.2 (7.9)	28.0 (8.1)
	Median (IQ range)					
Binge eating ^b^	8.0 (2–15) ^d^	1.0 (0–5.5) ^e^	0 (0–4.5) ^e^	0.5 (0–5) ^e^	2.0 (0–9.3)	0 (0–5.0) ^e^
Driven exercise ^c^	0.5 (0–10)	0 (0–12)	0 (0–8)	0 (0–8)	0 (0–10)	0 (0–7)

^a^ Eating Disorder Examination Questionnaire, Global and subscale scores [28]; ^b^ Monthly frequency of objectively large binge eating episodes as defined in the DSM-IV [5] and/or subjective binge eating episodes as defined in the Eating Disorder Examination [26]; ^c^ monthly frequency, ^d,e^ superscripts indicate a significant difference between means.

### 3.3. Discussion

The present study followed two cohorts of women with disordered eating over five years. The participants in the present study predominately had a problem with recurrent episodes of binge eating, either subjectively or objectively large, where they described their eating as beyond their control with or without dietary restriction. Disordered eating was associated with psychological distress, extreme concerns about weight, shape and eating and impaired mental health related quality of life. This was despite both groups receiving an intervention to prompt help-seeking either at the beginning or the end of the first year. However, help-seeking of any type for a problem related to eating disorder symptoms was not common in either cohort, ranging between 22 and 40% in any one year, and detailed interviews of Cohort 1 in the fifth year of the study indicated that few had accessed effective or evidence based therapy during the five years and if they did so it was mainly for weight loss and not for disordered eating [24]. Although there were high levels of dietary restraint and weight concern, BMI did not reduce over time. 

In both cohorts there appeared to be general symptomatic improvement in eating disorder symptoms and behaviours within the first year. However, after that the initial improvements waned. Time trends of eating disorder features were similar in the two cohorts with the exception of driven exercise. (In Cohort 1 there was significant fluctuation in frequency of driven exercise whereas there was no significant change in frequency found in Cohort 2.) It was unclear how much the initial general improvement may have been due to the intervention or effects of study participation irrespective of the group. At the end of the first year all participants had received the intervention and the findings for the RCTs were modest and mixed [21,22]. Improvement in general psychological distress did occur but was very small. Why time trends for frequency of driven exercise differed between cohorts and had the most fluctuating course of any behavior in the first cohort is unclear. However, the assessment of exercise by self-report is problematic and unlike other eating disorder behaviours, exercise has positive health attributes and social regard.

The findings differ from others, e.g., [15,16] which have suggested EDNOS and similar eating disorders may follow a benign course and/or high degree of symptom fluctuation over time [35]. These differences may be due in part to the nature of the sample recruitment (clinic *vs*. community) and selection (response to advertisement versus generally population survey) and the present study findings cannot be generalised to samples with clinically diagnosed eating disorder. Rather, possibly because this is a young adult community based sample the findings are more like the McKnight longitudinal naturalistic study and Abebe *et al.*, which found persistence of symptoms over time [17,18]. These findings highlight the importance of increasing access to and participation in effective treatments. Further research is needed to investigate the factors predictive of outcome, including treatment effects and the nature of psychopathology.

The strengths of the study are that participants in the first cohort were recruited directly from the community and whilst the second was a convenience sample, the two cohorts did not differ on key demographic or clinical features. The main limitation was the low rate of continuous participation at each year of follow-up. This is in spite of the 5-year retention rate of 64% in Cohort 1 and 52% in Cohort 2. We did consider data imputation but elected to present the data without imputation as the two cohorts had very similar findings and thus we present a study and replication study within the one research paper. It is possible that those who did not attend at each year would have differed in pattern of symptom severity, for example showing more fluctuation, more, or less improvement. However, there was no indication from baseline comparisons that their outcomes would differ in any predictable way. The low prevalence of purging type weight control behaviours in the present samples also precluded investigation of the persistence (or otherwise) of this eating disorder feature. Further limitations are the assessment of disordered eating by self-report (which may bias toward over-reporting binge eating [25] and limits generalisability to those with clinic diagnosed eating disorders) and the assessment of women only when men are also likely to suffer problems with recurrent binge eating and EDNOS [1,2].

## 4. Conclusions

Common eating disorder behaviours and psychopathology are associated with persistent general psychological distress and impairment in health related quality of life over time. For people with disordered eating, body weight is impervious to dietary restraint despite high levels of concern. This is likely because of concomitant irregular eating patterns and recurrent binge eating of sufferers. 

## Implications

More research is needed on how to assist people avail themselves of evidence based treatments such as cognitive behavior therapy. Without effective intervention disordered eating continues to be problematic.
